# Pelvic Ectopic Kidney in an Adult: Robotic-Assisted Surgical Treatment of Unrecognized Ureteropelvic Junction Obstruction

**DOI:** 10.1155/cris/6022407

**Published:** 2025-07-07

**Authors:** Amr Ahmed, Aleksa Zubelic, Milan Radovanovic, Gjoko Stojanoski, Jerome Katz

**Affiliations:** ^1^Department of Urology, Varisano Kliniken Frankfurt-Main-Taunus, Bad Soden, Germany; ^2^Clinic of Urology, University Clinical Centre of Serbia, Belgrade, Serbia

**Keywords:** pelvic kidney, robotic surgery, ureteropelvic junction obstruction

## Abstract

Ureteropelvic junction obstruction (UPJO) is observed in approximately 30% of patients with ectopic kidneys. Due to the narrow pelvic space and risk of injuring aberrant structures, an ectopic pelvic kidney with UPJO presents a unique treatment challenge. Most experiences in treating UPJO in pelvic ectopic kidneys using robotic surgical systems are based on the pediatric population. Only a few cases of successful robotic-assisted surgery in adult patients with this condition have been described. This case reports illustrates that the indications for robotic-assisted surgery for UPJO may safely be expanded to include complex adult cases with pelvic ectopic kidney.

## 1. Introduction

While pelvic kidneys represent the most common form of renal ectopia, they remain a rare anatomical variation, occurring in approximately 0.08% of the general population [1]. Although typically asymptomatic and often discovered incidentally, pelvic kidneys can be associated with other conditions such as nephrolithiasis, ureteropelvic junction obstruction (UPJO), and extrarenal calices. The renal pelvis in pelvic kidney is often located anteriorly, and is accompanied by a tortuous ureteral course as well as high insertion of ureter resulting in acute angulation at the ureteropelvic junction, leading to functional obstruction and hydronephrosis [2]. UPJO is observed in 22%–37% of patients with ectopic kidneys [3]. Common symptoms include intermittent abdominal pain, nausea, vomiting, and recurrent pyelonephritis, usually triggered by increased fluid intake and diuresis. Due to the narrow pelvic space and the increased risk of injuring aberrant vessels, overlying abdominal viscera, or nerves, UPJO in an ectopic pelvic kidney presents a unique treatment challenge [4]. Diagnosis of UPJO relies on dynamic renal scintigraphy using radiotracers such as ^99 m^Tc-DTPA or ^99 m^Tc-MAG3. However, the presence of structural and anatomical abnormalities necessitates additional cross-sectional imaging, with magnetic resonance (MR) urography being an excellent modality, particularly in younger patients [5]. The advantages of robotic surgery in treatment of UPJO are well established, and in cases involving UPJO in pelvic ectopic kidney, the benefits of the robot-assisted approach are even more apparent. Nevertheless, most reported experiences involve pediatric patients, while experiences with adult patients are limited. This case reports illustrates that the indications for robotic-assisted surgery for UPJO may safely be expanded to include complex adult cases, such as those involving a pelvic ectopic kidney.

## 2. Case Report

**Figure 1:**
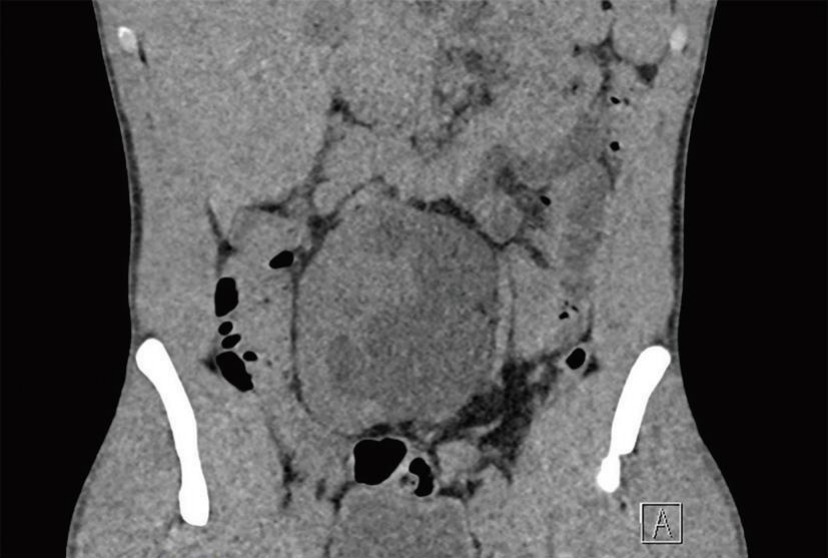
CT-abdomen (native low dose) showcasing ectopic malrotated pelvic right kidney with largely intrarenal renal pelvis and accompanying hydronephrosis.

**Figure 2:**
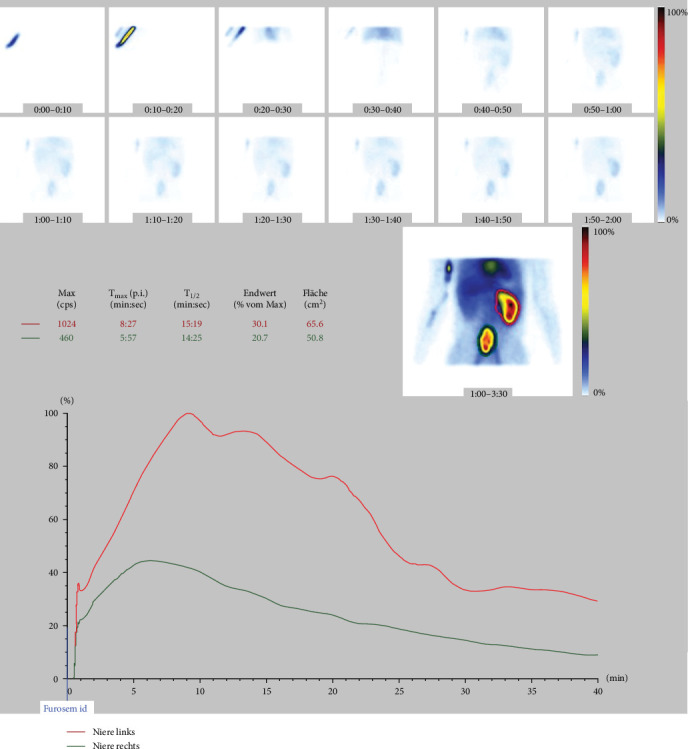
MAG3 scintigraphy showing prolonged clearance of the radiotracer, which, along with the clinical presentation, sonographic findings, and MRI scans, further supported the clinically suspected diagnosis of UPJO of the right kidney.

**Figure 3:**
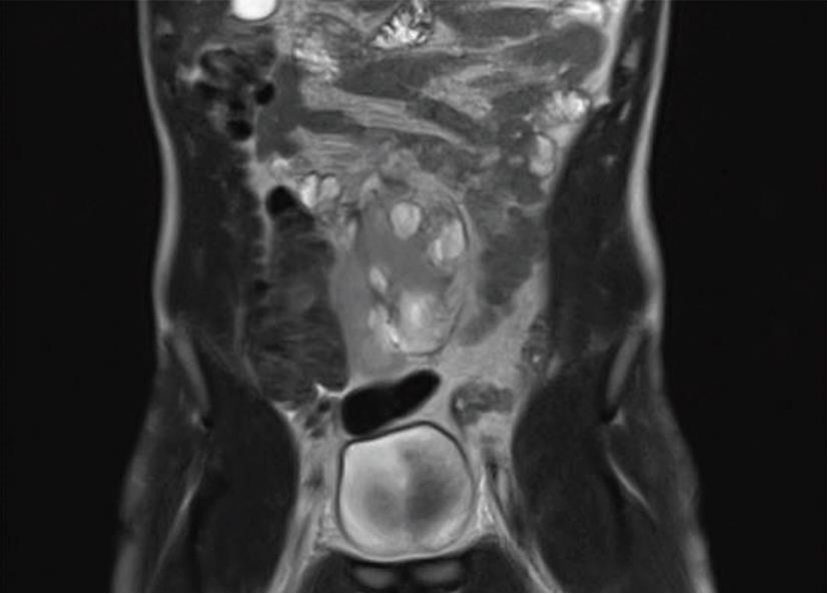
Magnetic resonance imaging-ectopic pelvic right kidney.

**Figure 4:**
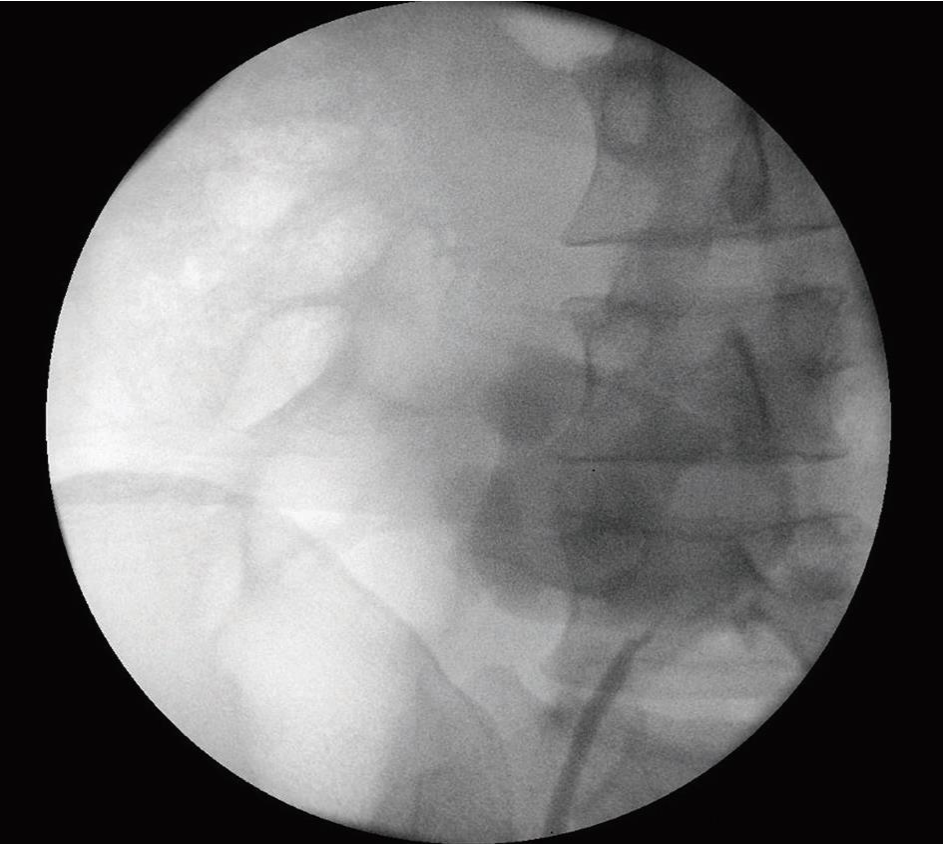
Retrograde urography during double-*J* stent implantation-left oriented ureter with high ureter insertion and accompanying UPJO.

A 22-year-old patient presented to our clinic with chronic and intermittent lower abdominal pain, often accompanied by nausea and vomiting. The symptoms were particularly pronounced after the intake of large volumes of fluid. Clinical examination and the complete blood count were unremarkable. An abdominal sonography was performed, revealing a pelvic localization of the right kidney with marked Grade III hydronephrosis. To rule out the relatively common concomitant urolithiasis associated with such cases, a native low-dose computed tomography (CT) was performed. It confirmed the presence of an ectopic, malrotated right pelvic kidney with a ventrally positioned and largely intrarenal renal pelvis, accompanied by hydronephrosis (Figure 1). The patient was referred for renal scintigraphy for further diagnostic and preoperative planning. MAG3 scintigraphy confirmed the clinical suspicion of a symptomatic UPJO (Figure 2). Due to the frequent presence of accompanying vascular and anatomical anomalies in such patients, further imaging with MRI was performed as part of the perioperative evaluation (Figure 3). During the ambulatory diagnostic process, the patient returned to our center with an acute episode of severe pain, nausea, and fever. Laboratory tests revealed a significant inflammatory response, with marked leukocytosis (white blood cell count of 17.8 x 10⁹/L) and elevated C-reactive protein levels (118.6 mg/L). Acute obstructive pyelonephritis was suspected. A catheterized urine sample was obtained for culture, which confirmed an *Escherichia coli* infection. Empirical antibiotic therapy was initiated and subsequently validated by antibiogram results. Simultaneously, a decision was made to implant a double-*J* ureteral stent. Images from the accompanying retrograde urography are shown in Figure 4.

**Figure 5:**
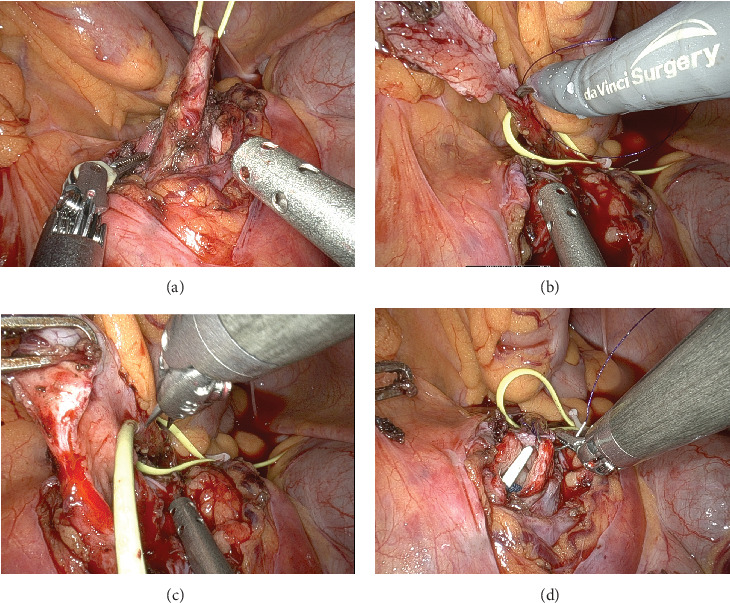
Intraoperative findings: (a) ureter dissection, (b) removal of fibrous excessive tissue, (c) double-*J* stent change, and (d) Anderson–Hynes pyeloplasty.

Following normalization of laboratory parameters and total convalescence of the patient, da Vinci-assisted robotic pyeloplasty was performed. Intraoperatively, due to the low position of ectopic kidney in the pelvis, a ventrally located renal pelvis, and a high, left-oriented ureteral insertion, a transmesenteric approach was used. Fibrous tissue strip overlying the ureteropelvic junction was dissected to allow optimal access. After successful resection of the UPJO, the existing 6 Fr double-*J* stent was replaced. An Anderson–Hynes (dismembered) pyeloplasty was then performed successfully (Figure 5). Indocyanine green (ICG) was extensively used throughout the procedure to identify any aberrant blood vessels, and to assess the viability of the ureteral tissue. The operative console time was 60 min, with an estimated blood loss of 50 mL. Postoperative creatinine and estimated glomerular filtration rate (eGFR) remained within normal limits. The patient was discharged on postoperative Day 4, and the double-*J* stent was removed after 8 weeks. At the 12-week postoperative follow-up, sonographic evaluation revealed a significant reduction in hydronephrosis. However, as expected in cases of long-standing obstruction, residual fixed caliectasis with minimal dilation of several calyces persisted. The patient reported complete resolution of symptoms, confirming the success of the treatment.

## 3. Discussion

The term pelvic kidney encompasses a range of anatomical abnormalities resulting from the kidney's failure to ascend from the pelvis during the metanephric stage of embryogenesis [6]. As the kidney ascends, its blood supply is derived from successive transient aortic branches. This ascent may be restricted if these transient blood vessels are not fully degenerated [7]. The reported incidence of pelvic kidney ranges from 2 to 10 per 10,000 individuals, depending on the method of detection. In rare cases, crossed or even fused ectopias may occur [8]. Ectopic kidneys are also associated with several other congenital abnormalities, such as VACTERL malformations and CHARGE syndrome [9]. In most cases, a pelvic kidney is an incidental finding and remains asymptomatic. Asymptomatic cases require monitoring, whereas in patients with recurrent symptoms—most often caused by the presence of UPJO, as observed in our case-timely surgical intervention is warranted [10]. UPJO is a well-recognized clinical entity that results in impaired urine flow from the renal pelvis into the ureter. If not detected and treated, it can ultimately lead to complete loss of renal function in the affected kidney. Most cases of UPJO are successfully treated surgically during childhood. Given the rarity of simultaneous UPJO and renal anomalies in the adult population—as seen in our case—there is a paucity of literature addressing surgical outcomes in this specific subset of patients [10]. The presence of hydronephrosis on sonography is usually the first indicator of UPJO; however, its presence does not necessarily reflect ongoing high-grade obstruction, and does not always correlate with clinical symptoms. The frequent co-occurrence of urolithiasis and aberrant vessels highlights the need for cross-sectional contrast imaging. The anatomical complexity of ectopic kidneys often renders traditional two-dimensional imaging modalities such as CT or MRI, insufficient for conveying the detailed anatomical information required for optimal surgical planning. As a result, 3D virtual reconstructions are gradually becoming the standard in preoperative preparation for complex cases [9]. Three-dimensional (3D) virtual reconstruction was not performed in our case due to insurance limitations. The absence of this imaging modality remains a major limitation in our preoperative planning. Robotic-assisted laparoscopic pyeloplasty (RAP) offers unique advantages in reconstructive surgery, including enhanced 3D visualization and improved precision in tissue handling and suturing. RAP has gained widespread popularity for pyeloplasty in both pediatric and adult populations [10]. Lukkanawong et al. [11] reported that the robotic approach can reduce the total operating time, drain removal time, and intraoperative blood loss compared to standard laparoscopic approaches. However, most reported experiences with RAP are based on the pediatric patients, and data on adult patients—particularly those with associated congenital anomalies—remain limited. When comparing surgical outcomes, laparoscopic and robotic approaches have shown similar success rates in various studies. However, the robotic approach is associated with lower postoperative complication and re-intervention rates. In contrast, a major disadvantage of the robotic approach appears to be significantly longer operating times. Some authors theorize that this may be due to selection bias, where more complex cases—especially in pediatric populations—are preferentially performed using the robotic method. A more objective limitation of the robotic approach remains its limited institutional availability and significantly higher operating costs, which often make robotic treatment of UPJO economically inaccessible in many countries [12–14]. Traditionally, transperitoneal laparoscopic access to the ureteropelvic junction has been performed via a retrocolic route. However, recent studies have demonstrated that the transmesenteric approach reduces operative time by an average of 22.5% and decreased hospital stay by 19.2%. This was also the approach used in our case [15]. Classical dismembered pyeloplasty has been used extensively utilized, with excellent and reproducible outcomes, and is considered the first-line choice for most patients. In our case, an Anderson–Hynes pyeloplasty—which has demonstrated an overall success rate of over 96.6% in various studies—was performed without complications [16, 17]. Postoperative outcomes of pyeloplasty in ectopic kidneys can be variable. One study reported hydronephrosis improvement in only 52.6% cases, often attributed to anatomy-related pelvocaliectasis. However, all relevant studies as well as our own results demonstrated significant symptomatic improvement following surgery [18, 19], thereby, supporting the necessity of timely surgical intervention.

## 4. Conclusion

A hydronephrotic pelvic kidney with UPJO presents a unique treatment challenge. Experience with adult patients affected by this anomaly is limited, highlighting the need for further studies to optimize treatment strategies for this population. However, as demonstrated in our case report, the availability of robotic surgical technology allows for the safe expansion of indications for minimally invasive pyeloplasty to include difficult adult cases, such as those with ectopic kidney position. This approach offers excellent symptom control and a low postoperative morbidity rate.

## Data Availability

The data that support the findings of this study are available upon request from the corresponding author. The data are not publicly available due to privacy or ethical restrictions.
